# Historical Exposure to Artificial Light at Night Shapes *Daphnia* Responses: An Experiment Across an Urban–Rural Gradient

**DOI:** 10.1002/ece3.72624

**Published:** 2025-12-11

**Authors:** Yuhan He, Huan Zhang, Jiale Guan, Panpan Wang, Yue Wu, Kangshun Zhao, Ulrika Candolin

**Affiliations:** ^1^ Key Laboratory of Lake and Watershed Science for Water Security, Key Laboratory of Breeding Biotechnology and Sustainable Aquaculture, Xiangxi River Ecosystem Research Station in the Three Gorges Reservoir Region, Institute of Hydrobiology Chinese Academy of Sciences Wuhan China; ^2^ Organismal and Evolutionary Biology University of Helsinki Helsinki Finland; ^3^ College of Fisheries Huazhong Agricultural University Wuhan China

**Keywords:** light pollution, phenotypic plasticity, reproduction, urbanization, zooplankton

## Abstract

Artificial light at night (ALAN) is an increasingly pervasive environmental stressor, yet its long‐term impacts on organisms are poorly understood. We used *Daphnia pulicaria* to investigate how historical exposure to ALAN influences life‐history and morphological responses when exposed to ALAN under laboratory conditions. The experiment spanned three generations and included clonal populations from historically exposed and unexposed lakes. Our results show that historically exposed populations have longer lifespans and clutch intervals than unexposed populations, irrespective of the presence or absence of laboratory ALAN. In the presence of laboratory ALAN, individuals from historically exposed populations mature earlier and produce more offspring, whereas individuals from unexposed populations show no changes in maturation or offspring production but have reduced spine length. Under laboratory ALAN, all populations have shorter clutch intervals and lifespans, reduced reproductive success, and smaller eyes and body sizes. Interestingly, the negative effect of laboratory ALAN on reproductive success intensifies across generations for historically unexposed populations, whereas the effect weakens for exposed populations. These findings indicate that historically exposed populations have partially adapted to ALAN whereas unexposed populations respond maladaptively and suffer reduced reproductive success across generations. This emphasizes the need to explore the evolutionary impacts of light pollution, its ecological consequences, and the potential of organisms to adapt to the stressor.

## Abstract 2

Artificial light at night (ALAN) is an increasingly pervasive environmental stressor, yet its long‐term impacts on organisms are poorly understood. We compared clonal populations from urban lakes (historically exposed to ALAN) and rural lakes (historically unexposed) under laboratory ALAN conditions. We found that historically exposed populations show degree of adaptation to ALAN, while unexposed populations respond maladaptively, suffering reduced reproductive success across generations.

## 1 Introduction

Artificial light at night (ALAN) is a form of human‐induced light pollution that brightens the night environment (Bara and Falchi 2023). Unlike natural light, ALAN disrupts the natural light–dark cycles to which organisms have adapted over evolutionary time, and which they depend on for critical biological processes (Gaston et al. 2017). This disruption affects behavior, physiology, and life‐history traits, with consequences for population dynamics (Lockett et al. 2021; Ganguly and Candolin 2023; Kivelä et al. 2023; Yang et al. 2024). For example, ALAN alters foraging patterns of amphipods (Lynn et al. 2021), interferes with reproductive strategies in glow‐worms (Elgert et al. 2020), and alters predation risks for various invertebrates (Czarnecka et al. 2019; Moyse et al. 2023). With the expansion of urban areas and the increasing use of artificial light (Kyba et al. 2017; Sánchez de Miguel et al. 2021), ALAN has become a pervasive environmental issue with significant implications for ecological systems and their functions (Hölker et al. 2010; Sanders et al. 2021).

Freshwater habitats are increasingly exposed to ALAN as urban areas are common around rivers and lakes (Kummu et al. 2011; Hölker et al. 2023). The habitats are exposed to direct illumination from terrestrial light sources, such as streetlights and buildings, and to skyglow (Jechow and Hölker 2019; Hölker et al. 2023). The artificial light disrupts the physiology and behavior of aquatic organisms, such as melatonin production, circadian rhythms, and feeding patterns (Bassi et al. 2021). These disruptions can alter species interactions and consequently influence broader ecological dynamics (Ganguly and Candolin 2023). Yet, most research on the impact of light pollution has focused on terrestrial organisms, while freshwater ecosystems have received comparatively little attention (Hölker et al. 2023).

Species in urban areas that have been exposed to ALAN for many generations might show some degree of adaptation to the light, depending on the strength of selection and the presence of genetic variation in the direction of selection (Candolin 2024). For instance, moths from light‐polluted areas show reduced flight‐to‐light behavior compared to those from unpolluted areas, although the possibility of developmental effects at the site of collection could not be excluded (Altermatt and Ebert 2016). Adaptation to ALAN could allow species to persist in light‐polluted environments, as found for several other human‐induced stressors (Sunday et al. 2014; Meester et al. 2018). However, the ability of species to adapt to light pollution is poorly known.

The crustaceans *Daphnia* spp. are keystone species of freshwater ecosystems that control algae biomass, serve as food for higher trophic levels, and recycle nutrients (Karuthapandi and Rao 2016). Many *Daphnia* species are sensitive to light conditions as they migrate from deeper water to surface water to feed during nights when predation risk is low (Hays 2003; Ekvall et al. 2020). These diel migrations can be disrupted by artificial light, which can reduce food intake (Moore et al. 2000; Maszczyk et al. 2021). Artificial light can also influence food selection (Cremer et al. 2022), anti‐predator defenses, and reproductive success (Li et al. 2022). Despite these disruptions, the ability of *Daphnia* species to adapt to ALAN is unknown. The negative effects on foraging, anti‐predator behavior, and reproduction suggest that ALAN could be a strong selection pressure. Responses to light pollution have been found to be clone‐specific (Maszczyk et al. 2021), which indicates the presence of genetic variation in responses to ALAN and, hence, the possibility of genetic adaptation. Given the ecological importance of *Daphnia* and their cosmopolitan distribution, more information is needed on the impact of ALAN on their activities and the ability to adapt to it.

The aim of this study was to investigate (1) if populations of *Daphnia pulicaria* historically exposed to ALAN have adapted to the light in terms of life history and morphology; and (2) if adaptation includes the evolution of plasticity. To address these questions, we exposed *D. pulicaria* from several urban and rural lakes, which have differed in ALAN exposure for several decades, to ALAN under laboratory conditions. We employed a crossed design and tracked three generations of clonal lines to assess whether responses changed across generations.

## 2 Materials and Methods

### 2.1 Experimental Populations

We collected *Daphnia pulicaria* from six lakes in Wuhan, China, one clone from each lake. Three of these were urban lakes that have been exposed to light pollution for over 40 years, with nighttime light intensities at the lake surface reaching approximately 10 lx (0.20 μmol cm^−2^ s^−1^, measured with a Li‐1500 light sensor). And three rural lakes were unexposed to light pollution (light intensity: < 0.001 lx, < 0.0001 μmol cm^−2^ s^−1^). See Appendix File S1 for detailed information on animal acquisition and lake characteristics (Table S1).

We cultivated the six clonal populations in the laboratory for at least 1 month to obtain enough individuals for the experiment. Rural populations were maintained under ambient light conditions and urban populations under ALAN, using different incubators to avoid light leakage. The photoperiod followed the natural photoperiod in the lakes (~13L:11D) using programmable broad‐spectrum LEDs. Daytime light levels were approximately 25 μmol cm^−2^ s^−1^ (~1087 lx), matching the light intensity at the lake surfaces. Rural populations experienced no artificial illumination during nights (< 0.0001 μmol cm^−2^ s^−1^, < 0.001 lx), while urban populations were exposed to ALAN (~0.20 μmol cm^−2^ s^−1^, ~10 lx). All populations were cultivated at 22°C in COMBO medium (Kilham et al. 1998) and fed *Chlorella* sp. (1.5 mg/L carbon). We renewed the water every 2 days to avoid waste accumulation.

### 2.2 Experimental Procedures

We used a 2 × 2 experimental design to examine the effects of historical ALAN (urban and rural populations) and of laboratory ALAN (presence or absence of ALAN) on fitness‐related traits. The light levels used in laboratory treatments were the same as during cultivation (absence of ALAN: < 0.001 lx, and presence of ALAN: 10 lx). The photoperiod followed the local natural cycle (~12L:12D). The experiment spanned three generations to assess plastic responses across generations.

To initiate the experiment, we randomly selected approximately 20 parthenogenetic individuals of similar body size from each of the six clonal populations, referred to as generation G0 (120 individuals in total). We cultivated them individually in 80 mL of COMBO medium under the same conditions as they had experienced during pre‐experimental cultivation, including light conditions (presence or absence of ALAN). We checked the cultivations every 12 h and collected neonates from the second clutch, generation G1, within 12 h of hatching. These G1 neonates were then divided between the two light treatments by randomly selecting 52 neonates from each of the six G1populations and assigned 26 neonates to the treatment with laboratory ALAN, and 26 to the treatment without laboratory ALAN. This resulted in 12 groups (six populations × two light treatments) with 26 individuals in each of the 12 groups (312 G1 individuals in total) (Figure S1). The individuals were cultivated individually in beakers with 80 mL of COMBO medium and food until the last individual had died (a maximum of 48 days).

### 2.3 Life‐History and Morphological Traits

To record age at maturity, we monitored the 12 groups of G1 individuals twice a day to assess the development of eggs in their brood pouches (Figure S2). We fixed six randomly selected mature individuals from each of the 12 groups in acid Lugol's solution for morphological measurements. We measured body length, body width, posterior spine length, and eye diameter, following (McKnight et al. 2023). The remaining 20 individuals (out of the 26) in each of the 12 groups were monitored once a day to record their life‐history traits: (1) age at release of first clutch of neonates or eggs, and time interval between clutches, (2) number of neonates in each clutch, and (3) age at death. We recorded the total number of offspring produced as a measure of lifetime reproductive success, excluding eggs that failed to hatch within the brood pouch.

### 2.4 Cultivation Across Generations

To assess changes in the measured traits across generations, we repeated the experiment for two additional generations. We randomly selected two neonates that had hatched within 24 h from the second clutches of each group and reared them under the same conditions as the parental generation. We repeated the measurements of traits as described above for these G2 and G3 individuals, that is, for 6 individuals for morphological traits and for up to 20 individuals for life‐history traits for each of the 12 groups (number of individuals per group is given in Table S2).

### 2.5 Statistical Analysis

All statistical analyzes were performed using R v. 4.4.1 (https://www.r‐project.org/). We checked correlations among life‐history and morphological traits (Figure S3) and removed traits that were strongly correlated with other traits (Correlation coefficient > 0.7) (Dormann et al. 2013). Retained life‐history traits were age at maturity, clutch interval, total number of offspring produced, and lifespan. Retained morphological traits were eye diameter, body length, and posterior spine length.

To assess the impact of historical and laboratory ALAN on the traits across the three generations, we used generalized linear mixed models (GLMMs). Fixed factors were historical ALAN, laboratory ALAN, and generation. Random factors were clonal population (nested within historical ALAN) and cultivation beaker. We ran normality tests to select the appropriate error distributions. We used GLMMs with a Gaussian distribution to analyze effects of the fixed factors on morphological traits and lifespan, GLMM with gamma distribution to analyze effects on age at maturity and clutch interval, and GLMM with Poisson distribution to analyze effects on total number of offspring produced (count data). We included all factors and interactions between fixed factors in initial models, and iteratively removed nonsignificant interaction terms (when *p* > 0.05) when this was supported by the Akaike information criterion (AIC) and did not influence the significance of the main factors (Akaike 1998). The GLMMs were run using the glmmTMB package (Brooks et al. 2017). The goodness of fit was checked by inspecting the residuals using the DHARMa package (Hartig 2022). We evaluated the significance of the fixed factors through the analysis of deviance, that is, Type II Wald chi‐square test using the ANOVA function from the car package (Fox and Weisberg 2019). When significant interactions between fixed factors were detected, we assessed the impact of the main effects using Tukey's pairwise comparisons from the emmeans package (Russell 2024).

## 3 Results

### 3.1 Life‐History

**FIGURE 1:**
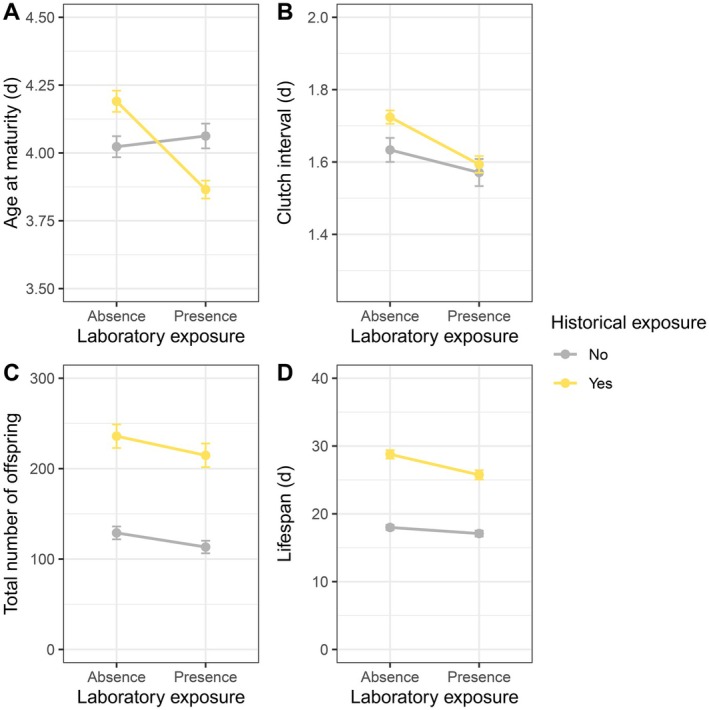
Effects of historical and laboratory ALAN on life‐history traits of *Daphnia pulicaria* across the three generations: (A) age at maturity; (B) clutch interval; (C) total number of offspring; (D) lifespan. Values are mean ± SE.

**FIGURE 2:**
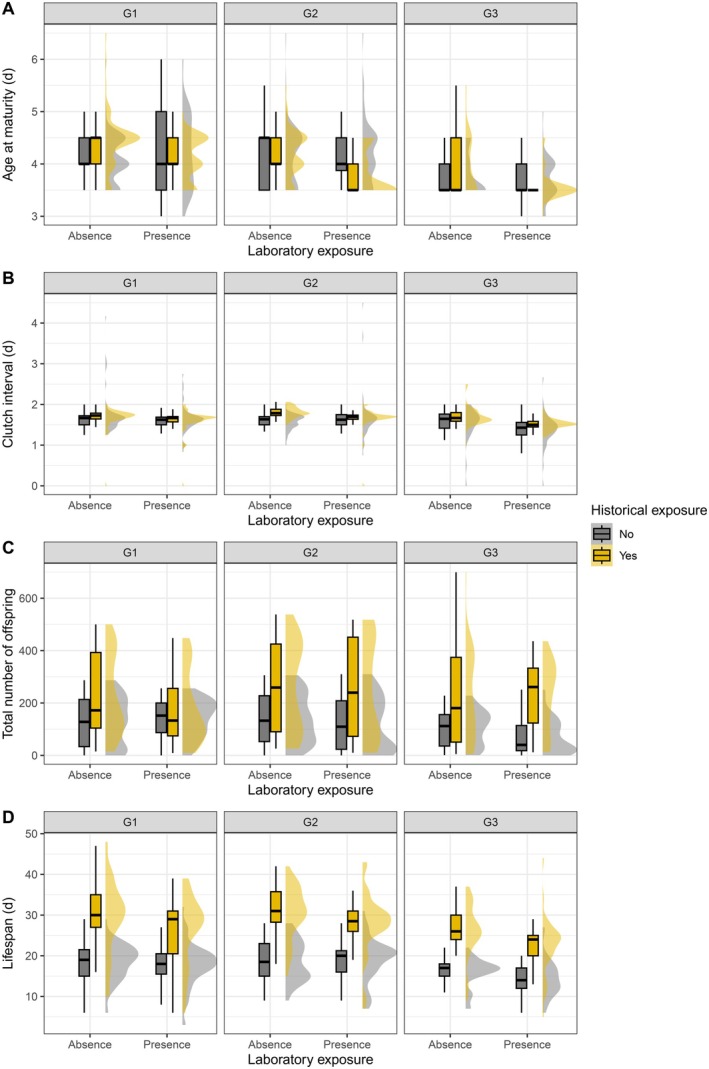
Cross‐generational effects of historical and laboratory exposure to ALAN on life‐history traits of *Daphnia pulicaria* : (A) age at maturity; (B) clutch interval; (C) total number of offspring; (D) lifespan. The boxplots show the median (horizontal line) and interquartile range (boxes). The half‐violin shapes depict the probability density of the data.

**FIGURE 3:**
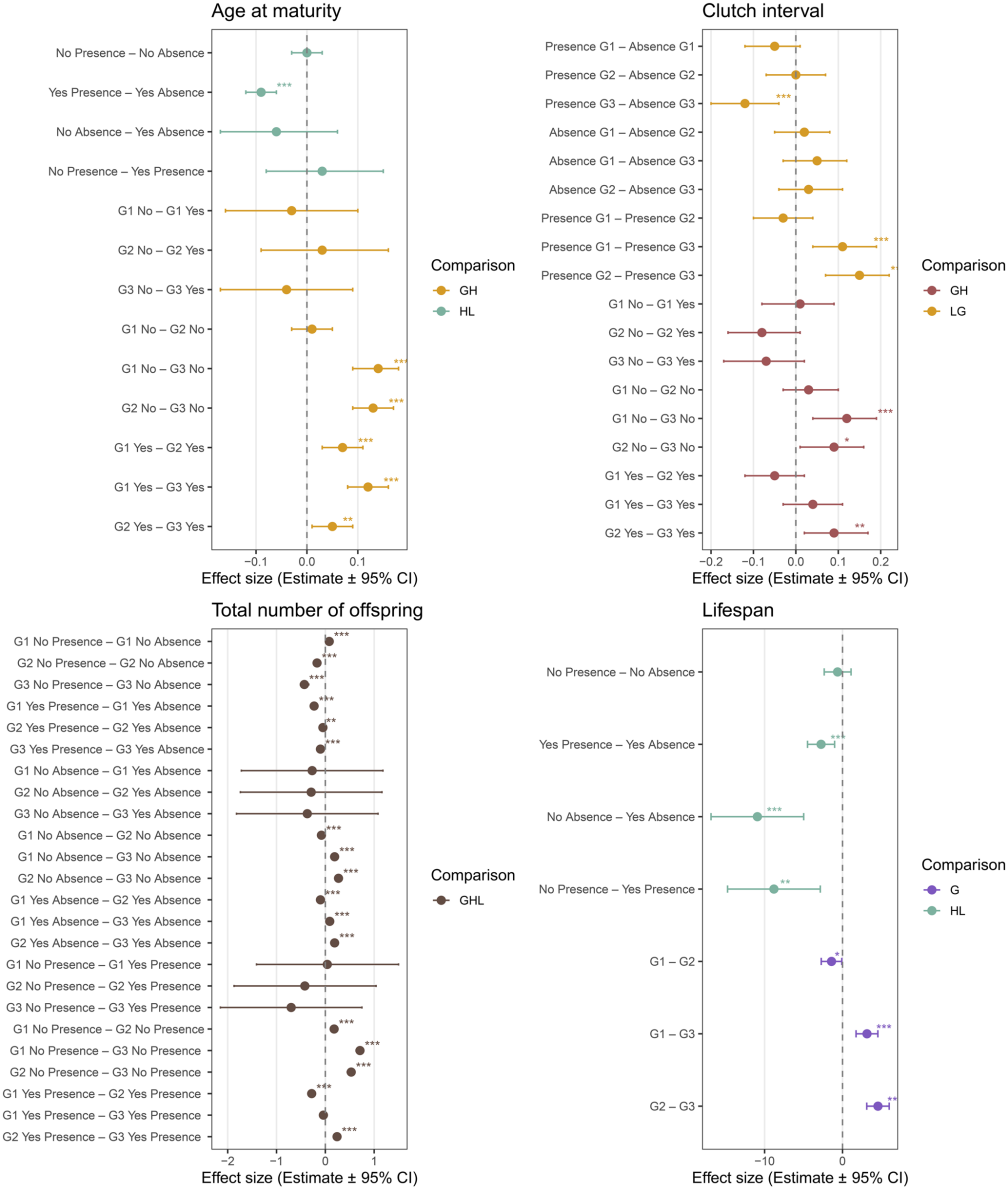
Effect sizes from Tukey's pairwise comparisons of historical (H) (Yes or No) and laboratory exposure (L) (Presence or Absence) to ALAN on life‐history traits of *Daphnia pulicaria* across generations (G) (G1, G2, and G3). Values are estimates ± 95% CI. Significant effects are indicated by asterisks (*p < 0.05, **p < 0.01, and ***p < 0.001, Table S3).

Historical ALAN influenced age at maturity depending on laboratory ALAN (Table 1, Figure 1A): individuals from historically exposed populations matured earlier under laboratory ALAN (Tukey's HSD, *z* = −7.51, *p* < 0.001), while unexposed populations did not differ in age at maturity between the presence and absence of laboratory ALAN (*z* = 0.36, *p* = 0.980). The magnitude of the difference between exposed and unexposed populations increased across generations, independent of laboratory ALAN: both matured earlier across generations (exposed: *z* = 8.87, *p* < 0.001; unexposed: *z* = 9.24, *p* < 0.001), but individuals from exposed populations matured earlier during the second generation (*z* = 5.25, *p* < 0.001), whereas unexposed populations matured earlier during the third generation (*z* = 8.60, *p* < 0.001) (Table S3, Figures 2A and 3).

**TABLE 1:** Effects of historical and laboratory exposure to ALAN on life‐history traits (age at maturity, clutch interval, total number of offspring produced, lifespan) and morphology (eye diameter, body length, and posterior spine length) of *Daphnia pulicaria* across three generations.

Error distribution	Response	Term	*X* ^2^	Df	*p*
Gamma	Age at maturity	Historical exposure	0.07	1	0.796
		Laboratory exposure	25.90	1	**< 0.001**
		Generation	164.11	2	**< 0.001**
		Historical × Laboratory	30.56	1	**< 0.001**
		Historical × Generation	16.48	2	**< 0.001**
Gamma	Clutch interval	Historical exposure	3.38	1	*0.066*
		Laboratory exposure	20.90	1	**< 0.001**
		Generation	11.52	2	**< 0.001**
		Historical × Generation	6.72	2	**0.035**
		Laboratory × Generation	7.34	2	**0.026**
Poisson	Total number of offspring produced	Historical exposure	0.36	1	0.546
		Laboratory exposure	319.98	1	**< 0.001**
		Generation	854.07	2	**< 0.001**
		Historical × Laboratory	2.52	1	0.113
		Historical × Generation	461.38	2	**< 0.001**
		Laboratory × Generation	33.93	2	**< 0.001**
		Historical × Laboratory × Generation	372.05	2	**< 0.001**
Gaussian	Lifespan	Historical exposure	19.12	1	**< 0.001**
		Laboratory exposure	12.48	1	**< 0.001**
		Generation	56.60	2	**< 0.001**
		Historical × Laboratory	4.97	1	**0.026**
Gaussian	Eye diameter	Historical exposure	0.01	1	0.932
		Laboratory exposure	6.48	1	**0.011**
		Generation	153.51	2	**< 0.001**
		Historical × Generation	10.17	2	**0.006**
Gaussian	Body length	Historical exposure	0.29	1	0.590
		Laboratory exposure	12.46	1	**< 0.001**
		Generation	26.77	2	**< 0.001**
		Laboratory × Generation	7.71	2	**0.021**
Gaussian	Posterior spine length	Historical exposure	2.87	1	*0.090*
		Laboratory exposure	8.17	1	**0.004**
		Generation	5.09	2	*0.078*
		Historical × Laboratory	9.21	1	**0.001**

*Note:* Generalized linear mixed models (GLMM) with different distributions were used to analyze the data. The table shows the significance of fixed effects by analysis of deviance, Type II Wald chi‐square tests. Bold numbers indicate *p* < 0.05, *italics* indicate 0.05 < *p* < 0.1.

Historical ALAN influenced the interval between clutches, with the effect depending on generation (Table 1, Figure 1B): the interval decreased across generations for historically unexposed populations (*z* = 4.55, *p* < 0.001) while it remained stable for exposed populations (*z* = 1.61, *p* = 0.590). Laboratory ALAN reduced clutch interval independent of historical ALAN but depending on generation (Table 1): the interval decreased across generations in the presence of laboratory ALAN (*z* = 4.44, *p* < 0.001), but not in the absence of it (*z* = 1.84, *p* = 0.440) (Table S3, Figures 2B and 3).

Historical ALAN influenced lifetime reproductive success depending on laboratory ALAN and generation (Table 1, Figure 1C): individuals from historically exposed populations produced fewer offspring in the presence of laboratory ALAN during all three generations (first: *z* = −16.86, *p* < 0.001; second: *z* = −3.86, *p* = 0.006; third: *z* = −6.65, *p* < 0.001), with offspring production increasing from generation 1 to 2 in the presence (*z* = −20.00, *p* < 0.001) and absence (*z* = −8.21, *p* < 0.001) of laboratory ALAN. In contrast, historically unexposed populations produced more offspring in the presence of laboratory ALAN during the first generation (first: *z* = 5.26, *p* < 0.001), but less during the second (*z* = −9.58, *p* < 0.001), and third generation (*z* = −16.65, *p* < 0.001), and their offspring production decreased across the three generations independent of laboratory ALAN (ALAN: *z* = 30.37, *p* < 0.001; Ambient: *z* = 9.09, *p* < 0.001) (Table S3, Figures 2C and 3).

Individuals from historically exposed populations had longer lifespans than those from unexposed populations and the difference was slightly larger in the absence of laboratory ALAN (Table 1, Figure 1D) (presence of ALAN: *t* = −3.81, *p* = 0.001; absence: *t* = −4.74, *p* < 0.001). Individuals from historically exposed populations had shorter lifespans under laboratory ALAN (*t* = −4.07, *p* < 0.001), whereas the lifespans of unexposed populations were unaffected by laboratory ALAN (*t* = −0.94, *p* = 0.786). Lifespan was shorter during the third generation than during the first (*t* = 5.27, *p* < 0.001) and second generation (*t* = 7.41, *p* < 0.001), independent of historical and laboratory ALAN (Table S3, Figures 2D and 3).

### 3.2 Morphology

**FIGURE 4:**
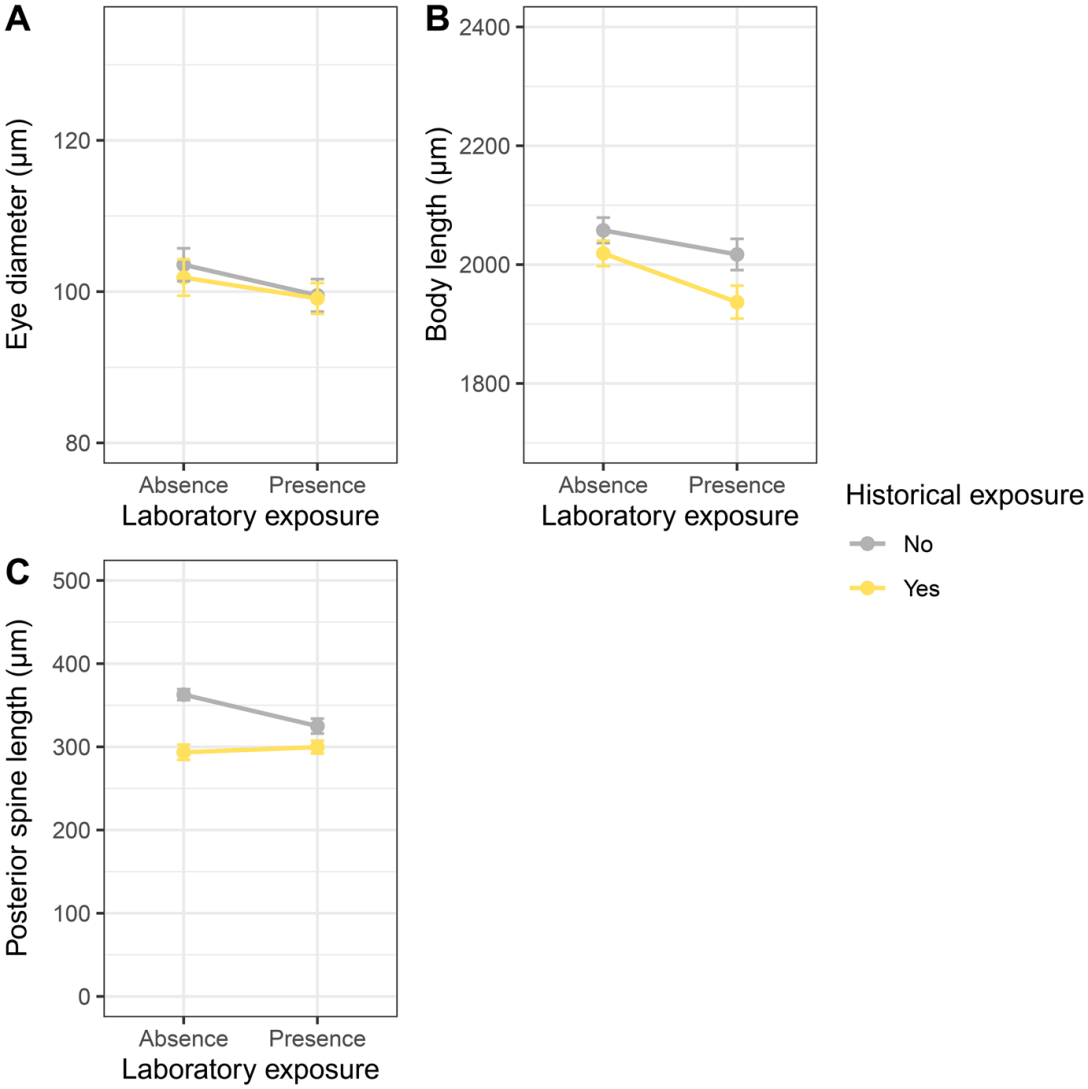
Effects of historical and laboratory ALAN on morphological traits of *Daphnia pulicaria* across the three generations: (A) eye diameter; (B) body length; (C) posterior spine length. Values are mean ± SE.

**FIGURE 5:**
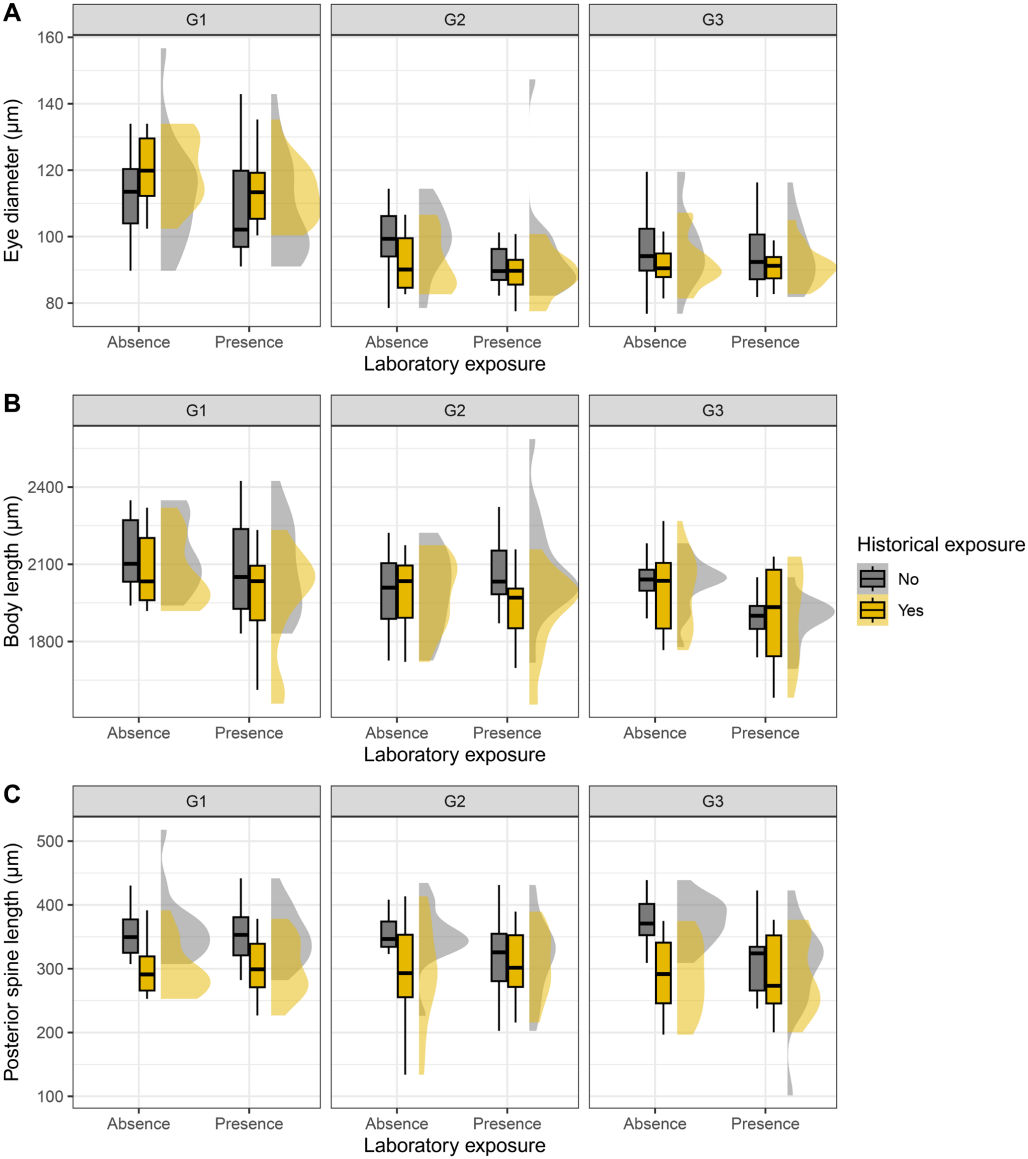
Cross‐generational effects of historical and laboratory exposure to ALAN on morphological traits of *Daphnia pulicaria* : (A) eye diameter; (B) body length; (C) posterior spine length. The boxplots show the median (horizontal line) and interquartile range (boxes). The half‐violin shapes depict the probability density of the data.

**FIGURE 6:**
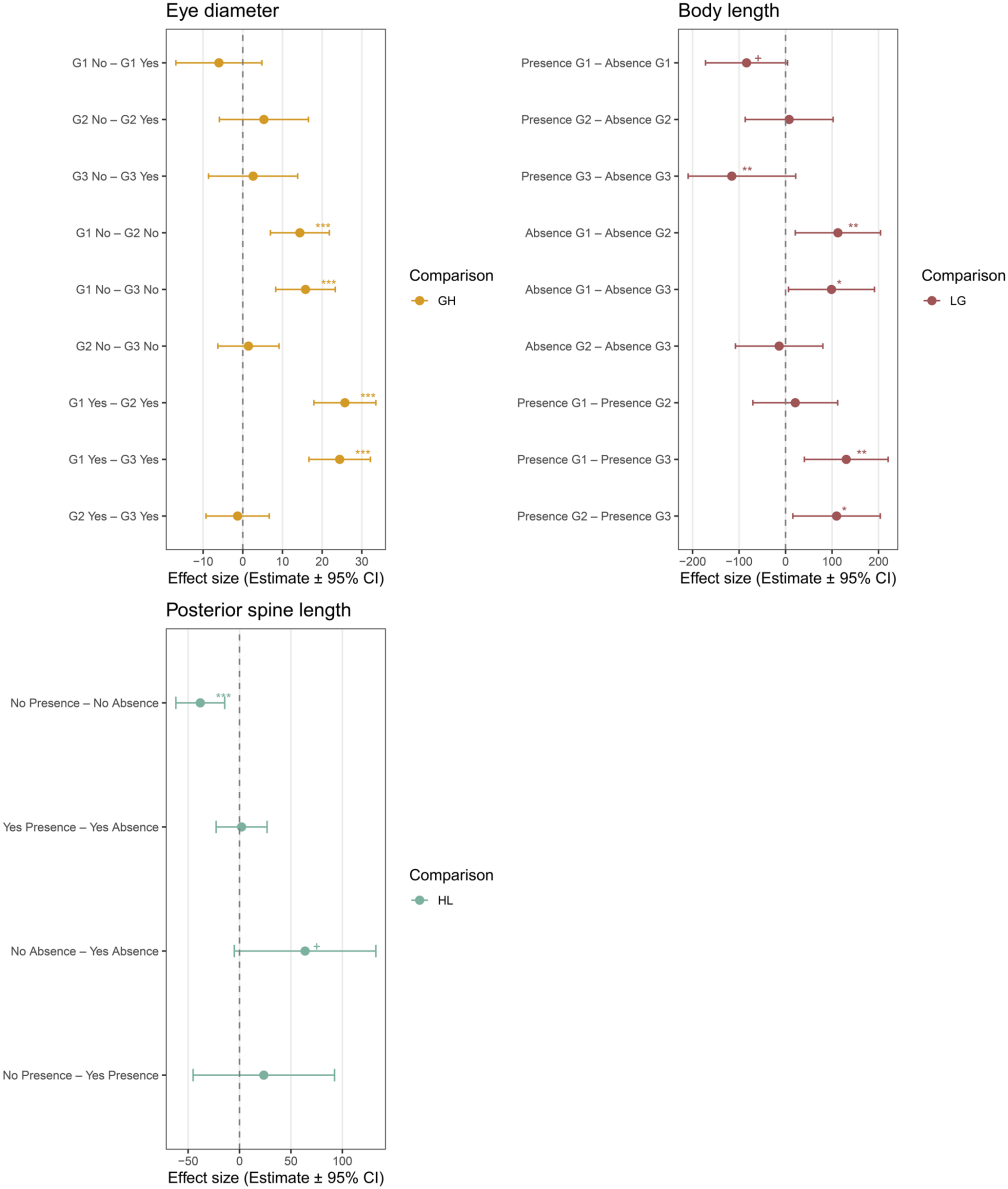
Effect size of historical (H) (Yes and No) and laboratory exposure (L) (Presence or Absence) to ALAN on morphological traits of *Daphnia pulicaria* across generations (G) (G1, G2, and G3) from Tukey's pairwise comparisons. Values are estimate ± 95% CI. Significant effects are indicated by asterisks (+*p* < 0.1, *p < 0.05, **p < 0.01, and ***p < 0.001, Table S4).

Laboratory ALAN reduced eye diameter independent of historical ALAN or generation (Table 1, Figure 4A). Eye diameter decreased across generations and the reduction was larger in historically exposed populations (*t* = 9.09, *p* < 0.001) than in unexposed populations (*t* = 6.07, *p* < 0.001) (Table S4, Figures 5A and 6).

Laboratory ALAN reduced body length depending on generation (Table 1): individuals under laboratory ALAN had shorter body length at maturity during the third generation (*t* = −3.55, *p* = 0.007) independent of historical exposure, and they tended to have shorter body length during the first generation (*t* = −2.74, *p* = 0.072), but not during the second generation (*t* = 0.24, *p* = 1.000). Pairwise comparisons further revealed that body length decreased across generations in the presence (*t* = 4.18, *p* = 0.001) and absence (*t* = 3.08, *p* = 0.029) of laboratory ALAN (Table S4, Figures 5B and 6).

Exposure to laboratory ALAN reduced posterior spine length depending on historical exposure (Table 1, Figure 4C): the length decreased in unexposed populations (*t* = −4.16, *p* < 0.001), while it did not change in exposed populations (*t* = 0.21, *p* = 0.997). The length decreased across generations independent of historical or laboratory ALAN (Table S4, Figures 5C and 6).

## 4 Discussion

The impact of ALAN on organisms is of growing concern, with scarce information about long‐term effects and the ability of species to adapt to the light (Hölker et al. 2023; Candolin 2024). Using a globally abundant and ecologically important freshwater zooplankton with a short generation time, *Daphnia pulicaria* , we found historically exposed urban populations to differ in life‐history traits from unexposed rural populations under common garden conditions, and to show signs of adaptation to ALAN when exposed to it under laboratory conditions.

Specifically, the results show that individuals from urban populations that have been exposed to ALAN for several decades have longer lifespans than individuals from unexposed rural populations, irrespective of the presence or absence of ALAN under laboratory conditions. They also grow faster, mature earlier, and produce more offspring than individuals from historically unexposed populations when exposed to ALAN in the laboratory. Moreover, while individuals from both exposed and unexposed populations have lower reproductive success when exposed to laboratory ALAN, the negative effect decreases across generations for exposed populations while it increases for unexposed populations.

These findings indicate that individuals in populations historically exposed to ALAN have adapted to ALAN to some degree, while individuals from unexposed populations respond maladaptively and suffer increasingly lower reproductive success across generations. Thus, exposure to ALAN in urban lakes appears to have driven local adaptation to the light, with selection strengthening across generations (Hopkins et al. 2018). Individuals with traits that confer higher fitness under ALAN, such as faster growth, earlier maturation, longer lifespan, and larger offspring production, may have passed more of their genes to the following generations, which, over time, may have increased these traits in the population, leading to a population‐wide shift in life‐history traits (Hopkins et al. 2018).

Yet, exposure to laboratory ALAN had negative effects on lifespan and reproductive success for both historically exposed and unexposed populations, although with more severe effects for unexposed populations. This indicates that the historically exposed, urban populations have not fully adapted to the light, despite several decades of ALAN. In the wild, light serves as a crucial environmental cue that regulates diel vertical migration with *Daphnia* retreating to deeper, darker waters during daylight to avoid predators (Gust et al. 2019). However, light pollution disrupts the diel rhythm, which affects food intake, food selection, anti‐predator defenses, behavior, and reproductive success (Moore et al. 2000; Maszczyk et al. 2021; Cremer et al. 2022; Li et al. 2022; Lehtonen and Candolin 2025). Hence, to fully ignore artificial light, or to adapt to it, may not be possible, as the light may increase predation risk and reduce food intake (Effertz and von Elert 2017).

Eye size and body length did not differ between historically exposed and unexposed populations, but both traits were smaller under laboratory ALAN, independent of historical exposure, and the traits further decreased in size across generations. This aligns with previous studies finding exposure to artificial light to limit body growth (Li et al. 2022) and reduce eye size in *Daphnia* (Brandon and Dudycha 2014). The reduction in body size may reflect a plastic response to increased perceived predation risk, with individuals maturing earlier—at a smaller size—to maximize lifetime reproductive success. Yet, historically unexposed populations exposed to laboratory ALAN developed shorter posterior spines although longer spines are expected to provide better protection from predators (Tollrian 1995). It is plausible that the reductions in morphological traits are energy‐saving adaptations, as maintaining large sensory and structural organs could be metabolically costly in constantly illuminated environments.

To further assess the impact of ALAN on zooplankton and the influence of the characteristics of the light, as well as the ability of populations to adapt to the light, future studies should include a wider range of light intensities and spectral distributions, as well as consider more populations. Moreover, more realistic environmental conditions, which include predators and the possibility of diel vertical migration, should be adopted to assess the limitations of adaptation (Tüzün et al. 2025).

To conclude, our results show that lake populations of a common zooplankton have partially adapted to ALAN during several decades of light pollution, but that the adaptation is incomplete with individuals suffering some fitness reductions when exposed to ALAN under laboratory conditions. This indicates that species that use darkness as a shelter against predators, such as many zooplankton, may have difficulties adapting to ALAN. Thus, to maintain biodiversity and ecosystem functions, we need to reduce light pollution by lowering light intensities and restricting the temporal and spatial use of light at night (Gaston 2018; Jechow et al. 2020; Owens et al. 2020; Hölker et al. 2023).

## Author Contributions

**Yuhan He:** conceptualization (equal), data curation (equal), formal analysis (lead), funding acquisition (supporting), investigation (equal), methodology (lead), resources (lead), software (lead), validation (equal), visualization (lead), writing – original draft (lead), writing – review and editing (equal). **Huan Zhang:** funding acquisition (lead), methodology (equal), project administration (equal), validation (equal), writing – review and editing (equal). **Jiale Guan:** data curation (equal), formal analysis (equal), investigation (equal). **Panpan Wang:** data curation (equal), formal analysis (equal), investigation (equal). **Yue Wu:** formal analysis (equal), investigation (equal). **Kangshun Zhao:** data curation (supporting), formal analysis (supporting), investigation (supporting). **Ulrika Candolin:** conceptualization (equal), project administration (equal), supervision (lead), writing – review and editing (equal).

## Funding

This research was funded by the China Scholarship Council (to Y.H.), the National Natural Science Foundation of China (Grant 32171515) and the Jiangxi Provincial Key Research and Development Program (Grant 20243BBH81037) (to H.Z.).

## Conflicts of Interest

The authors declare no conflicts of interest.

## Supporting information


**Data S1:** ece372624‐sup‐0001‐DataS1.xlsx.

**Data S2:** ece372624‐sup‐0002‐DataS2.xlsx.

**File S1:** ece372624‐sup‐0003‐Supinfo.docx.

## Data Availability

The data that supports the findings of this study is available in the Supporting Information of this article.
