# Supplementary material for: Historical Exposure to Artificial Light at Night Shapes Daphnia Responses: An Experiment Across an Urban–Rural Gradient
Source: Ecol Evol. 2025 Dec 11;15(12):e72624. doi: 10.1002/ece3.72624 (PMC12696422; doi:10.1002/ece3.72624)
Supplement: Supplementary file 3 — File S1: ece372624‐sup‐0003‐Supinfo.docx. [file ECE3-15-e72624-s003.docx]

## Supporting information for

**Historical exposure to artificial light shapes *Daphnia* responses: an experiment across an urban-rural gradient**

**Populations**

Based on light pollution levels (<https://darkmap.cn/>) and water quality (<http://lake.geodata.cn/>), we collected *Daphnia pulicaria* in three rural lakes (light levels during cloudy nights measured in December 2023 using Li-1500 light sensor logger: <0.001 lx, <0.0001 μmol cm^−2^ s^−1^) and three urban lakes that have been exposed to ALAN for several decades (light intensity: 10 lx, 0.20 μmol cm^−2^ s^−1^) (Table S1). To maximize the number of *Daphnia pulicaria* populations collected, we collected both live zooplankton from the water column and resting eggs from the top layer of lake sediment between December 2023 and January 2024. These samples were brought back to lab for isolating or hatching, cultivation and identification.

**Experiment animal identification**

We cultivated the isolated or newly hatched individuals from different lakes individually in separate beakers containing COMBO medium (Kilham *et al.* 1998), and fed them with *Chlorella* sp. Once each clone reaches a population of more than 10 individuals, we fixed one adult individual from each clone population in ethanol for morphological identification to identify putative *D. pulicaria.* Subsequently, we used one individual from each putative *Daphnia pulicaria* population and obtained their SSU sequences for accurate species identification (Montero-Pau, Gómez & Muñoz 2008).

**Table S1** Lakes from which *Daphnia pulicaria* were obtained

| **Lakes** | **Latitude** | **Longitude** | **Light environment** |
| --- | --- | --- | --- |
| #ZSH | 30.1395 | 114.1960 | Ambient |
| #XSH | 30.1403 | 114.1737 | Ambient |
| #LH | 30.1949 | 114.2307 | Ambient |
| #NSH | 30.5625 | 114.3163 | ALAN |
| #WHG | 30.5937 | 114.2832 | ALAN |
| #HZH | 30.5976 | 114.2840 | ALAN |

**Replicates used in experiment**

For Generation G1, we used 26 individuals per group, and from each generation (except G3), we randomly selected two neonates from the second clutch (hatched within 12h) and reared them under the same conditions as their mother (Figure S2). Not all individuals produced neonates in the second clutch, see final replicates in Table S2.

**Table S2** Number of replicates for each generation for each of the 12 groups

| **Generation** | **Laboratory ALAN** | **Historical ALAN** | **Population** | **Replicates** |
| --- | --- | --- | --- | --- |
| G1 | Presence | Exposed | #HZH | 26 |
|  |  |  | #WHG | 26 |
|  |  |  | #NSH | 26 |
|  |  | Not exposed | #LH | 26 |
|  |  |  | #ZSH | 26 |
|  |  |  | #XSH | 26 |
|  | Absence | Exposed | #HZH | 26 |
|  |  |  | #WHG | 26 |
|  |  |  | #NSH | 26 |
|  |  | Not exposed | #LH | 26 |
|  |  |  | #ZSH | 26 |
|  |  |  | #XSH | 26 |
| G2 | Presence | Exposed | #HZH | 26 |
|  |  |  | #WHG | 17 |
|  |  |  | #NSH | 20 |
|  |  | Not exposed | #LH | 23 |
|  |  |  | #ZSH | 26 |
|  |  |  | #XSH | 12 |
|  | Absence | Exposed | #HZH | 26 |
|  |  |  | #WHG | 26 |
|  |  |  | #NSH | 20 |
|  |  | Not exposed | #LH | 25 |
|  |  |  | #ZSH | 23 |
|  |  |  | #XSH | 20 |
| G3 | Presence | Exposed | #HZH | 26 |
|  |  |  | #WHG | 14 |
|  |  |  | #NSH | 16 |
|  |  | Not exposed | #LH | 23 |
|  |  |  | #ZSH | 24 |
|  |  |  | #XSH | 9 |
|  | Absence | Exposed | #HZH | 25 |
|  |  |  | #WHG | 22 |
|  |  |  | #NSH | 19 |
|  |  | Not exposed | #LH | 19 |
|  |  |  | #ZSH | 18 |
|  |  |  | #XSH | 8 |

**Figure S1** Experimental design


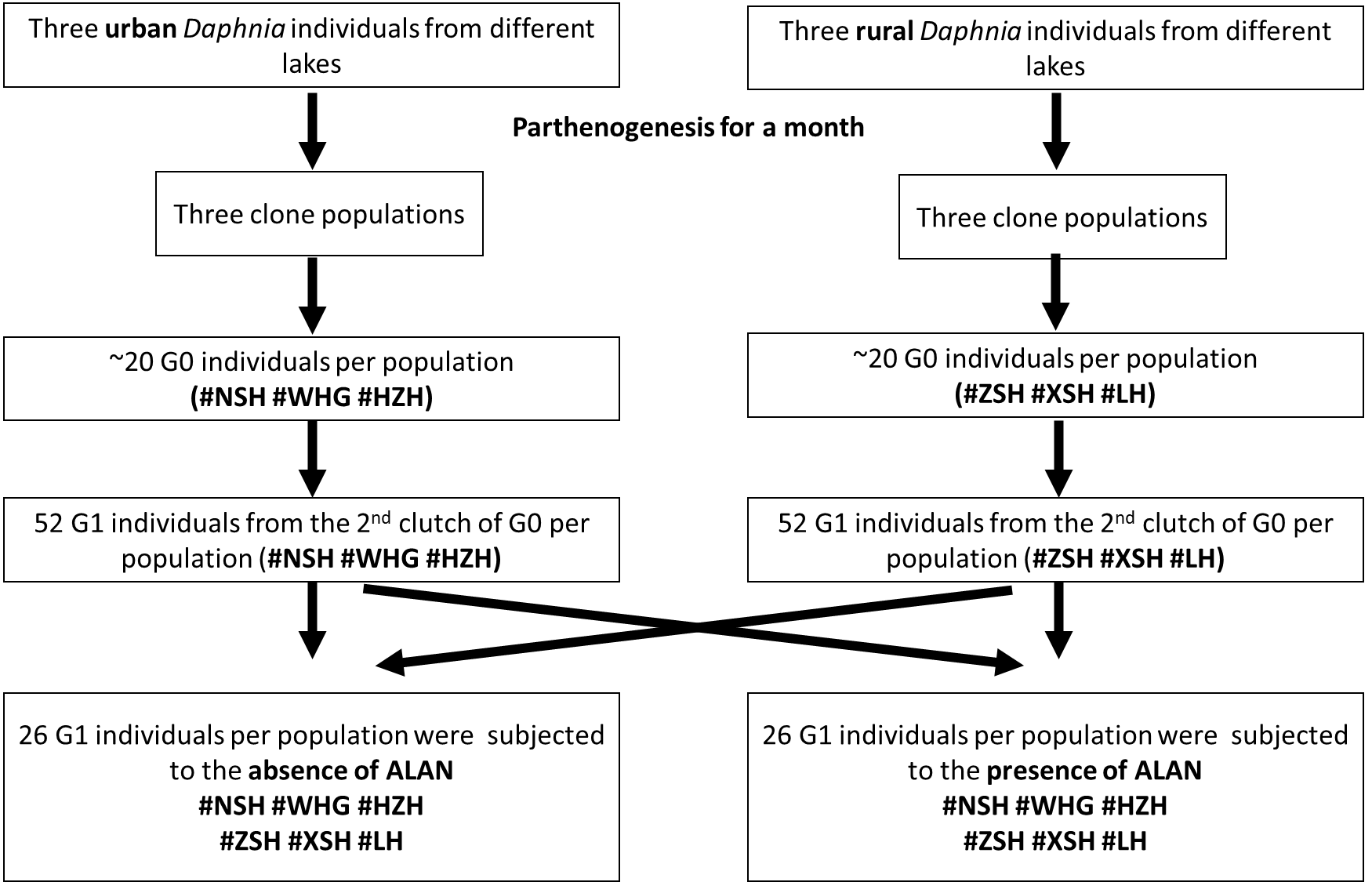


**Figure S2** Matured *Daphnia pulicaria* with parthenogenetic eggs in brood pouch


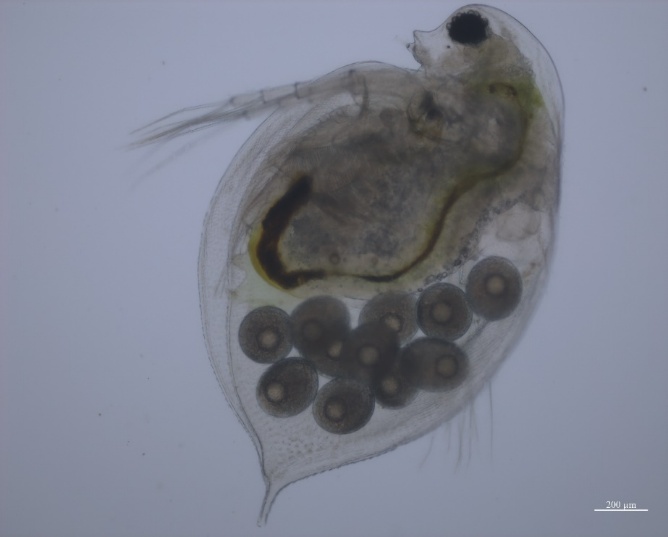


**Figure S3** Correlation coefficiant plot was used to diagnoise colinearity between life-history traits and remove traits with values > 0.7.


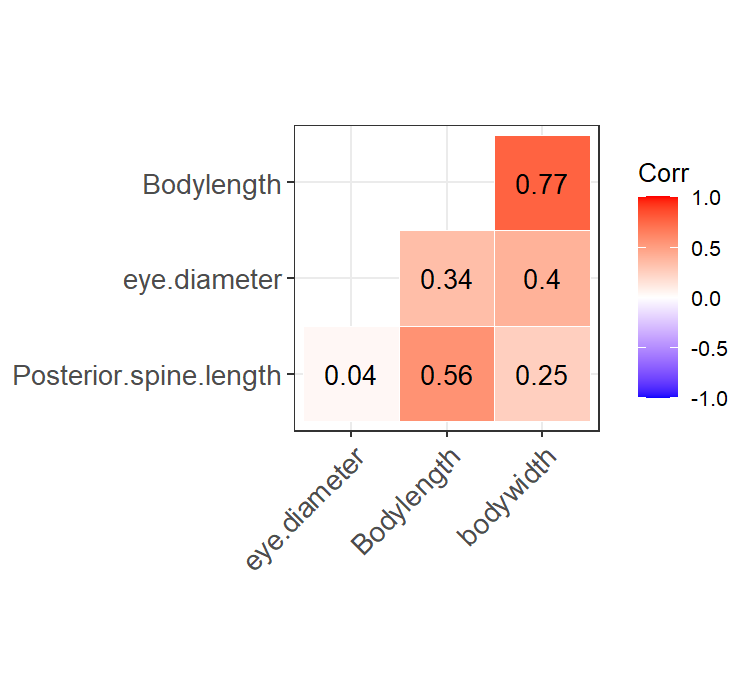

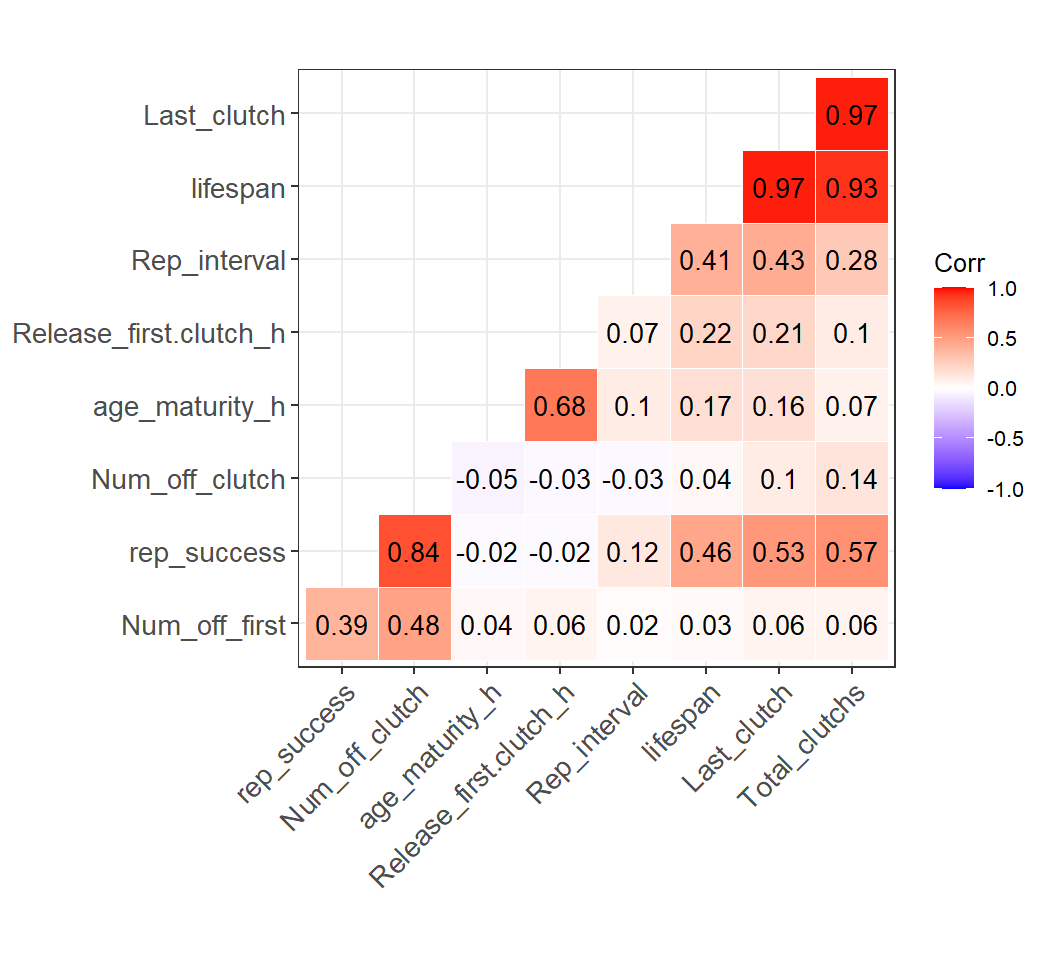


**Table S3** Tukey’s pairwise comparisons between historical exposure (Yest or No), laboratory exposure (Presence or Absence), and generation (G1, G2, or G3). (A) Age at maturity; (B) Clutch interval; (C) Total number of offspring produced; (D) Lifespan. Bold numbers indicate P < 0.05, *italics* indicate 0.05 < p < 0.1.

**A**

| **Response** | | **Contrast** | **SE** | **z ratio** | **P value** | **Estimate** | **Lower 95% CI** | **Upper 95% CI** |
| --- | --- | --- | --- | --- | --- | --- | --- | --- |
| Age at maturity | No Presence – Yes Presence | | 0.04 | 0.73 | 0.886 | 0.03 | -0.08 | 0.15 |
|  | No Presence – No Absence | | 0.01 | 0.36 | 0.985 | 0.00 | -0.03 | 0.03 |
|  | No Absence – Yes Absence | | 0.04 | -1.29 | 0.569 | -0.06 | -0.17 | 0.06 |
|  | Yes Presence – Yes Absence | | 0.01 | -7.51 | **<0.001** | -0.09 | -0.12 | -0.06 |
|  | G1 No - G2 No | | 0.01 | 0.56 | 0.993 | 0.01 | -0.03 | 0.05 |
|  | G2 No - G3 No | | 0.02 | 8.60 | **<0.001** | 0.13 | 0.09 | 0.17 |
|  | G1 No - G3 No | | 0.01 | 9.24 | **<0.001** | 0.14 | 0.09 | 0.18 |
|  | G1 No - G1 Yes | | 0.04 | -0.66 | 0.986 | -0.03 | -0.16 | 0.10 |
|  | G2 No - G2 Yes | | 0.05 | 0.75 | 0.976 | 0.03 | -0.09 | 0.16 |
|  | G3 No - G3 Yes | | 0.05 | -0.91 | 0.943 | -0.04 | -0.17 | 0.09 |
|  | G1 Yes - G2 Yes | | 0.01 | 5.25 | **<0.001** | 0.07 | 0.03 | 0.11 |
|  | G1 Yes - G3 Yes | | 0.01 | 8.87 | **<0.001** | 0.12 | 0.08 | 0.16 |
|  | G2 Yes - G3 Yes | | 0.01 | 3.72 | **0.003** | 0.05 | 0.01 | 0.09 |

**B**

| **Response** | **Contrast** | **SE** | **z ratio** | **P value** | **Estimate** | **Lower 95% CI** | **Upper**  **95% CI** |
| --- | --- | --- | --- | --- | --- | --- | --- |
| Clutch interval | G1 No - G2 No | 0.02 | 1.40 | 0.726 | 0.03 | -0.03 | 0.10 |
|  | G2 No - G3 No | 0.03 | 3.17 | **0.019** | 0.09 | 0.01 | 0.16 |
|  | G1 No - G3 No | 0.03 | 4.55 | **<0.001** | 0.12 | 0.04 | 0.19 |
|  | G1 No - G1 Yes | 0.03 | 0.20 | 1.000 | 0.01 | -0.08 | 0.09 |
|  | G1 Yes - G2 Yes | 0.02 | -2.06 | 0.311 | -0.05 | -0.12 | 0.02 |
|  | G1 Yes - G3 Yes | 0.03 | 1.61 | 0.590 | 0.04 | -0.03 | 0.11 |
|  | G2 No - G2 Yes | 0.03 | -2.58 | 0.102 | -0.08 | -0.16 | 0.01 |
|  | G2 Yes - G3 Yes | 0.03 | 3.51 | **0.006** | 0.09 | 0.02 | 0.17 |
|  | G3 No - G3 Yes | 0.03 | -2.22 | 0.230 | -0.07 | -0.17 | 0.02 |
|  | G1 Presence - G2 Presence | 0.02 | -1.37 | 0.745 | -0.03 | -0.10 | 0.04 |
|  | G2 Presence - G3 Presence | 0.03 | 5.51 | **<0.001** | 0.15 | 0.07 | 0.22 |
|  | G1 Presence - G3 Presence | 0.03 | 4.44 | **<0.001** | 0.11 | 0.04 | 0.19 |
|  | G1 Presence - G1 Absence | 0.02 | -2.29 | 0.197 | -0.05 | -0.12 | 0.01 |
|  | G1 Absence - G2 Absence | 0.02 | 0.69 | 0.983 | 0.02 | -0.05 | 0.08 |
|  | G1 Absence - G3 Absence | 0.03 | 1.84 | 0.441 | 0.05 | -0.03 | 0.12 |
|  | G2 Presence - G2 Absence | 0.03 | -0.12 | 1.000 | 0.00 | -0.07 | 0.07 |
|  | G2 Absence - G3 Absence | 0.03 | 1.17 | 0.851 | 0.03 | -0.04 | 0.11 |
|  | G3 Presence - G3 Absence | 0.03 | -4.27 | **<0.001** | -0.12 | -0.20 | -0.04 |

**C**

| **Response** | **Contrast** | **SE** | **z ratio** | **P value** | **Estimate** | **Lower 95% CI** | **Upper 95% CI** |
| --- | --- | --- | --- | --- | --- | --- | --- |
| Total number of offspring produced | G1 No Presence - G2 No Presence | 0.02 | 9.72 | **<0.001** | 0.18 | 0.12 | 0.24 |
|  | G2 No Presence - G3 No Presence | 0.02 | 22.00 | **<0.001** | 0.53 | 0.45 | 0.60 |
|  | G1 No Presence - G3 No Presence | 0.02 | 30.37 | **<0.001** | 0.71 | 0.63 | 0.78 |
|  | G1 No Presence - G1 Yes Presence | 0.44 | 0.10 | 1.000 | 0.04 | -1.41 | 1.50 |
|  | G1 No Presence - G1 No Absence | 0.02 | 5.26 | **<0.001** | 0.08 | 0.03 | 0.13 |
|  | G1 No Absence - G1 Yes Absence | 0.44 | -0.60 | 1.000 | -0.27 | -1.72 | 1.18 |
|  | G1 No Absence - G2 No Absence | 0.02 | -4.82 | **<0.001** | -0.08 | -0.13 | -0.03 |
|  | G2 No Absence - G3 No Absence | 0.02 | 12.79 | **<0.001** | 0.27 | 0.20 | 0.34 |
|  | G1 No Absence - G3 No Absence | 0.02 | 9.09 | **<0.001** | 0.19 | 0.12 | 0.26 |
|  | G1 Yes Absence - G2 Yes Absence | 0.01 | -8.21 | **<0.001** | -0.10 | -0.15 | -0.06 |
|  | G2 Yes Absence - G3 Yes Absence | 0.01 | 14.62 | **<0.001** | 0.19 | 0.15 | 0.24 |
|  | G1 Yes Absence - G3 Yes Absence | 0.01 | 6.70 | **<0.001** | 0.09 | 0.05 | 0.13 |
|  | G2 No Presence - G2 Yes Presence | 0.44 | -0.94 | 0.999 | -0.42 | -1.87 | 1.04 |
|  | G2 No Presence - G2 No Absence | 0.02 | -9.58 | **<0.001** | -0.17 | -0.23 | -0.11 |
|  | G1 Yes Presence - G1 Yes Absence | 0.01 | -16.86 | **<0.001** | -0.23 | -0.27 | -0.18 |
|  | G2 Yes Presence - G2 Yes Absence | 0.01 | -3.86 | **0.006** | -0.05 | -0.09 | -0.01 |
|  | G3 Yes Presence - G3 Yes Absence | 0.02 | -6.65 | **<0.001** | -0.10 | -0.15 | -0.05 |
|  | G1 Yes Presence - G2 Yes Presence | 0.01 | -20.00 | **<0.001** | -0.28 | -0.33 | -0.24 |
|  | G2 Yes Presence - G3 Yes Presence | 0.01 | 16.43 | **<0.001** | 0.24 | 0.20 | 0.29 |
|  | G1 Yes Presence - G3 Yes Presence | 0.02 | -2.40 | 0.408 | -0.04 | -0.09 | 0.01 |
|  | G3 No Presence - G3 Yes Presence | 0.44 | -1.57 | 0.920 | -0.70 | -2.15 | 0.75 |
|  | G3 No Presence - G3 No Absence | 0.03 | -16.65 | **<0.001** | -0.43 | -0.51 | -0.34 |
|  | G2 No Absence - G2 Yes Absence | 0.44 | -0.66 | 1.000 | -0.29 | -1.74 | 1.16 |
|  | G3 No Absence - G3 Yes Absence | 0.44 | -0.83 | 1.000 | -0.37 | -1.82 | 1.08 |

**D**

| **Response** | **Contrast** | **SE** | **t ratio** | **P value** | **Estimate** | **Lower 95% CI** | **Upper 95% CI** |
| --- | --- | --- | --- | --- | --- | --- | --- |
| Lifespan | No Presence - Yes Presence | 2.31 | -3.81 | **0.001** | -8.80 | -14.75 | -2.85 |
|  | No Presence - No Absence | 0.67 | -0.94 | 0.786 | -0.63 | -2.35 | 1.10 |
|  | Yes Presence - Yes Absence | 0.68 | -4.07 | **<0.001** | -2.75 | -4.49 | -1.01 |
|  | No Absence - Yes Absence | 2.31 | -4.74 | **<0.001** | -10.92 | -16.87 | -4.98 |
|  | G1 - G2 | 0.56 | -2.51 | **0.033** | -1.41 | -2.73 | -0.09 |
|  | G1 - G3 | 0.60 | 5.27 | **<0.001** | 3.14 | 1.74 | 4.54 |
|  | G2 - G3 | 0.61 | 7.41 | **<0.001** | 4.55 | 3.11 | 5.99 |

**Table S4** Tukey’s pairwise comparison between historical exposure (Yest or No), experimental exposure (Presence or Absence), and generation (G1, G2, or G3) in GLMMs. (A) Eye diameter; (B) body length; (C) posterior spine length. Bold numbers indicate P < 0.05, *italics* indicate 0.05 < p < 0.1.

**A**

| **Response** | **Contrast** | **SE** | **t ratio** | **P value** | **Estimate** | **Lower 95% CI** | **Upper 95% CI** |
| --- | --- | --- | --- | --- | --- | --- | --- |
| Eye diameter | G1 No - G2 No | 2.58 | 5.58 | **<0.001** | 14.38 | 6.96 | 21.81 |
|  | G2 No - G3 No | 2.68 | 0.53 | 0.995 | 1.42 | -6.29 | 9.13 |
|  | G1 No - G3 No | 2.61 | 6.07 | **<0.001** | 15.80 | 8.30 | 23.31 |
|  | G1 No - G1 Yes | 3.76 | -1.60 | 0.599 | -6.02 | -16.87 | 4.82 |
|  | G2 No - G2 Yes | 3.89 | 1.37 | 0.747 | 5.32 | -5.90 | 16.55 |
|  | G3 No - G3 Yes | 3.90 | 0.67 | 0.985 | 2.60 | -8.64 | 13.83 |
|  | G1 Yes - G2 Yes | 2.71 | 9.49 | **<0.001** | 25.73 | 17.91 | 33.54 |
|  | G1 Yes - G3 Yes | 2.69 | 9.09 | **<0.001** | 24.42 | 16.68 | 32.16 |
|  | G2 Yes - G3 Yes | 2.77 | -0.47 | 0.997 | -1.31 | -9.28 | 6.66 |

**B**

| **Response** | **Contrast** | **SE** | **t ratio** | **P value** | **Estimate** | **Lower 95% CI** | **Upper 95% CI** |
| --- | --- | --- | --- | --- | --- | --- | --- |
| Body length | G1 Presence - G2 Presence | 31.70 | 0.66 | 0.986 | 20.86 | -70.42 | 112.10 |
|  | G2 Presence - G3 Presence | 32.60 | 3.36 | **0.012** | 109.68 | 15.64 | 203.70 |
|  | G1 Presence - G3 Presence | 31.30 | 4.18 | **0.001** | 130.55 | 40.45 | 220.60 |
|  | G1 Presence - G1 Absence | 30.60 | -2.74 | *0.072* | -83.95 | -172.20 | 4.30 |
|  | G2 Presence - G2 Absence | 32.80 | 0.24 | 1.000 | 7.74 | -86.80 | 102.28 |
|  | G3 Presence - G3 Absence | 32.60 | -3.55 | **0.007** | -115.70 | -209.60 | 21.77 |
|  | G1 Absence - G2 Absence | 31.80 | 3.54 | **0.007** | 112.56 | 20.86 | 204.30 |
|  | G2 Absence - G3 Absence | 32.60 | -0.42 | 0.998 | -13.77 | -107.83 | 80.30 |
|  | G1 Absence - G3 Absence | 32.10 | 3.08 | **0.029** | 98.79 | 6.33 | 191.30 |

**C**

| **Response** | **Contrast** | **SE** | **t ratio** | **P value** | **Estimate** | **Lower 95% CI** | **Upper 95% CI** |
| --- | --- | --- | --- | --- | --- | --- | --- |
| Posterior spine length | Presence No - Absence No | 9.16 | -4.16 | **<0.001** | -38.14 | -61.90 | -14.40 |
|  | Presence No - Presence Yes | 26.50 | 0.89 | 0.810 | 23.61 | -45.14 | 92.40 |
|  | Absence No - Absence Yes | 26.54 | 2.40 | *0.080* | 63.76 | -5.07 | 132.60 |
|  | Presence Yes - Absence Yes | 9.55 | 0.21 | 0.997 | 2.01 | -22.74 | 26.80 |

**Reference**

Kilham, S.S., Kreeger, D.A., Lynn, S.G., Goulden, C.E. & Herrera, L. (1998) COMBO: a defined freshwater culture medium for algae and zooplankton. *Hydrobiologia,* **377,** 147-159.

Montero-Pau, J., Gómez, A. & Muñoz, J. (2008) Application of an inexpensive and high‐throughput genomic DNA extraction method for the molecular ecology of zooplanktonic diapausing eggs. *Limnology and Oceanography: Methods,* **6,** 218-222.
